# Influence of Agronomic Practices on the Bioactive Compound Production in *Cannabis sativa* L.

**DOI:** 10.3390/ijms262210999

**Published:** 2025-11-13

**Authors:** Esperanza Dalmau, Mónica Umaña, Valeria Eim, José Bon, Susana Simal

**Affiliations:** 1 Department of Chemistry, University of the Balearic Islands, 07122 Palma, Spain; esperanza.dalmau@uib.es (E.D.); monica.umana@uib.es (M.U.); valeria.eim@uib.es (V.E.); 2 Analysis and Simulation of Agro-food Processes Group, Instituto Universitario de Ingeniería de Alimentos-FoodUPV, Universitat Politècnica de València, Camino de Vera s/n, 46022 Valencia, Spain; jbon@upvnet.upv.es

**Keywords:** *Cannabis sativa* L., cannabinoids, terpenes, antioxidants, cultivar, agronomic management, flowering time

## Abstract

Industrial hemp phytochemistry is shaped by genetics and agronomic management, yet field studies integrating both remain scarce. The combined effects of cultivar, planting density, fertilization, and flowering time on cannabinoids, terpenes, and antioxidants in *Cannabis sativa* L. were evaluated. A field trial was conducted in Mallorca (2023) using two cultivars (*Enectaliana*, *Enectarol*) grown at two densities (Sector 1 ≈ 2.3 plants m^−2^; Sector 2 ≈ 4.6 plants m^−2^), with sampling from flowering onset (week 0) to week 5. In *Enectaliana*, fertilization (with vs. without) was tested. *Enectaliana* displayed CBD/CBDVA-dominated profiles, whereas *Enectarol* was CBG-predominant; THC remained consistently low. Effects were assessed via three-way ANOVA (Density × Time × Cultivar; Density × Time × Fertilization). The cultivar and time explained most of the variance, with interactions modulating magnitudes without altering effect hierarchies. Planting density acted as a second-order modulator, modulating concentrations without reversing cultivar rankings. Terpenes peaked early and generally declined as flowering progressed, with cultivar-dependent trajectories. Total phenolics and antioxidant activity (ABTS, FRAP assays) increased steadily until week 5, with density and treatment effects. In *Enectaliana*, fertilization effects were selective: ABTS values tended to be higher in unfertilized plants at the end of the cycle, FRAP results showed a density interaction, and cannabinoids exhibited non-linear responses to nutrient supply.

## 1. Introduction

*Cannabis sativa* L., commonly known as hemp, is a plant from the Cannabaceae family that has been cultivated for centuries for its multiple industrial, medicinal, and recreational uses. Historically, hemp has been used for fibre, oil, and seed production, leveraging its properties as a strong and nutritious material. However, in recent decades, interest in hemp has significantly increased due to its content of bioactive compounds such as cannabinoids, terpenes, and antioxidants, which possess a wide range of therapeutic benefits and industrial applications [1].

Among cannabinoids, cannabidiol (CBD) and cannabigerol (CBG) have stood out for their medicinal properties. CBD is known for its anti-inflammatory, analgesic, and anxiolytic effects, without the psychoactive effects associated with tetrahydrocannabinol (THC), another cannabinoid present in hemp. CBG has shown potential as a neuroprotective and antimicrobial agent. These compounds are of great interest to the pharmaceutical and nutraceutical industries, as they can be used in the treatment of various conditions, from chronic pain to neurological disorders [2]. In addition to cannabinoids, terpenes present in hemp, such as α-pinene, limonene, myrcene, and β-caryophyllene, not only contribute to the characteristic aroma of the plant but also have therapeutic properties. Terpenes can interact synergistically with cannabinoids, enhancing their medicinal effects through the so-called “entourage effect” [3]. For example, myrcene is known for its sedative and analgesic properties, while limonene has anxiolytic effects and can improve mood. Furthermore, hemp also exhibits a high antioxidant compounds content that can protect cells from oxidative damage and contribute to cardiovascular and metabolic health. The concentration of these antioxidant compounds can vary significantly depending on the hemp variety and cultivation conditions, highlighting the importance of research in this field to maximize their benefits [4,5]

The genetic diversity of *Cannabis sativa* L. allows for the selection of specific varieties that produce different profiles of bioactive compounds, adapting to various industrial and medicinal applications. This genetic variability, combined with advanced cultivation techniques, offers the possibility of optimizing the production of specific compounds, such as CBD and CBG, based on market needs. Cultivars like *Enectaliana* and *Enectarol* are examples of this genetic diversity, standing out for their high contents of CBD and CBG, respectively. Although these cultivars were recently included in the catalogue of permitted cultivation varieties in Europe [6], there are no published studies on them yet.

The growing demand for hemp-derived products has driven the need for research to optimize agricultural practices to maximize the yield and quality of bioactive compounds. This includes evaluating factors such as planting density, fertilizer use, and flowering time, which can significantly influence the concentration of cannabinoids, terpenes, and antioxidant compounds. The interaction of these factors and their impact on the production of bioactive compounds remains a crucial area of study for the development of more efficient and sustainable cultivation strategies [1]. Fertilization is one of the most studied factors in hemp production. Previous research has shown that nutrient availability can significantly influence the biosynthesis of cannabinoids and terpenes. For instance, the application of organic or mineral fertilizers can boost the accumulation of cannabinoids such as CBD and THC [3]. Additionally, proper nutrition can also affect the terpene profile, increasing the production of compounds such as myrcene and limonene, which contribute to both the sensory and therapeutic properties of hemp [7]. Planting density also plays a crucial role in the production of bioactive compounds. Several studies indicate that increased competition for light, water, and nutrients at higher planting densities can alter the concentration of cannabinoids and other compounds [1]. An optimal planting density can maximize biomass yield and the accumulation of bioactive compounds, although the magnitude and direction of these effects may vary according to cultivar and environmental conditions [8]. Genetic variability is another determining factor in the production of bioactive compounds in hemp. Different studies have shown that the plant’s genetics can significantly impact the concentration of cannabinoids and terpenes [4]. These genetic differences not only affect the quantity of compounds produced, but also their profile and proportion, which is essential for specific applications in the pharmaceutical and nutraceutical industries. Flowering time is a key determinant of cannabinoids, terpenes, and antioxidant compounds accumulation. Longer flowering periods generally allow greater biosynthesis, resulting in higher concentrations of these compounds [9]. However, their levels fluctuate throughout the flowering stage, making harvest timing critical for maximizing bioactive content [5].

Despite abundant research on individual factors, there is a lack of studies considering the simultaneous interaction of multiple variables. Most research has focused on isolated factors, without evaluating how the combination of cultivar, flowering time, fertilizer use, and planting density can influence the overall production of bioactive compounds. This lack of comprehensive studies limits the ability to optimize cultivation practices to maximize the yield and quality of hemp for industrial and medicinal uses.

This study aims to fill this gap in existing literature by providing a comprehensive evaluation of how the combination of different agricultural practices affects the production of bioactive compounds in two cultivars of hemp (*Enectaliana* and *Enectarol*) for which the authors have not found previous published studies. The objective of the study was to investigate the combined effect of cultivar (*Enectaliana* and *Enectarol*), flowering time, fertilizer application, and planting density on the concentration of cannabinoids, terpenes, and antioxidants in *Cannabis sativa* L. It is expected to contribute to the development of more effective and sustainable cultivation strategies that maximize the benefits of *Cannabis sativa* L. for various industrial and medicinal applications.

## 2. Results and Discussion

**Figure 1:**
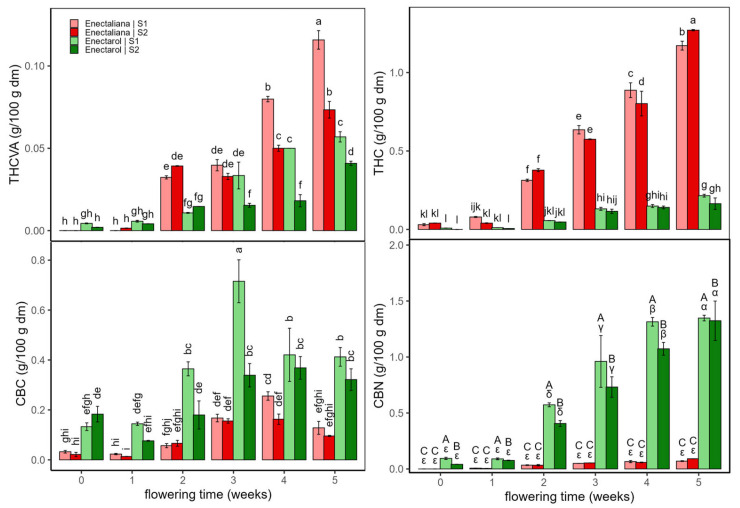
Psychoactive cannabinoids and related derivatives in *Enectaliana* and *Enectarol* cultivars from 0 to 5 flowering weeks for two planting densities (Sector 1 (S1) ≈ 2.3 plants m^−2^; Sector 2 (S2) ≈ 4.6 plants m^−2^): THCVA, THC, CBC and CBN. Different letters indicate significant differences (*p* < 0.05). Lowercase for Density × Time × Cultivar in THCVA, THC and CBC; uppercase (Density × Cultivar) and Greek (Time × Cultivar) in CBN.

**Figure 2:**
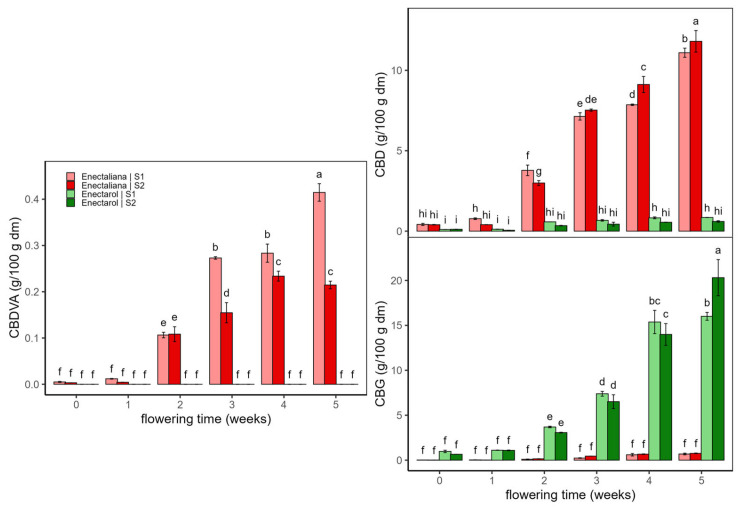
Non-psychoactive cannabinoids in *Enectaliana* and *Enectarol* cultivars from 0 to 5 flowering weeks for two planting densities (Sector 1 (S1) ≈ 2.3 plants m^−2^; Sector 2 (S2) ≈ 4.6 plants m^−2^): CBDVA, CBD and CBG. Different letters indicate significant differences (*p* < 0.05).

**Figure 3:**
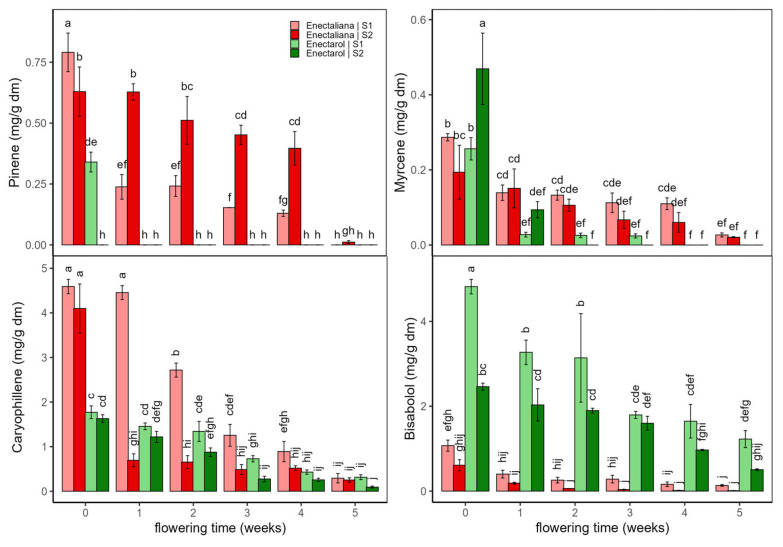
Terpene content (α-pinene, myrcene, caryophyllene, and bisabolol; mg/g dm) in *Enectaliana* and *Enectarol* cultivars from 0 to 5 flowering weeks for two planting densities (Sector 1 (S1) ≈ 2.3 plants m^−2^; Sector 2 (S2) ≈ 4.6 plants m^−2^). Different letters indicate significant differences (*p* < 0.05).

**Figure 4:**
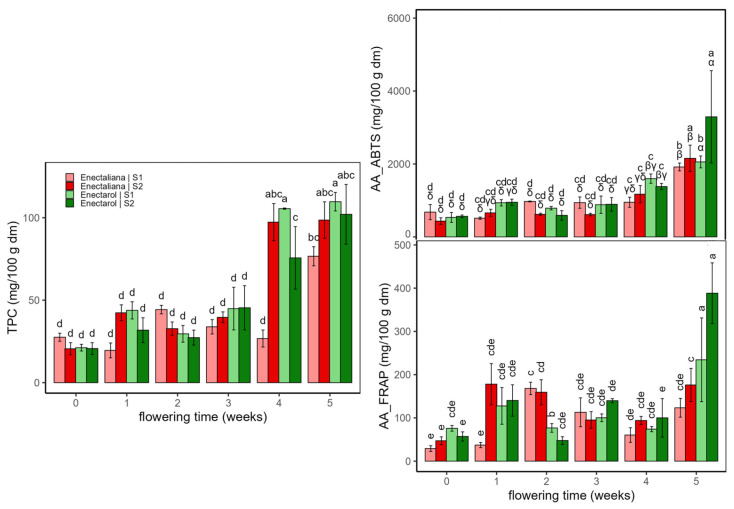
Total polyphenol content (TPC, mg GAE/100 g dm) and antioxidant activity (AA: ABTS and FRAP, mg Trolox/100 g dm) in *Enectaliana* and *Enectarol* cultivars from 0 to 5 flowering weeks for two planting densities (Sector 1 (S1) ≈ 2.3 plants m^−2^; Sector 2 (S2) ≈ 4.6 plants m^−2^). Different letters indicate significant differences (*p* < 0.05) between samples of different factors. Lowercase for Density × Time × Cultivar in TPC and AA_FRAP; uppercase (Density × Time) and Greek (Time × Cultivar) in AA_ABTS.

**Figure 5:**
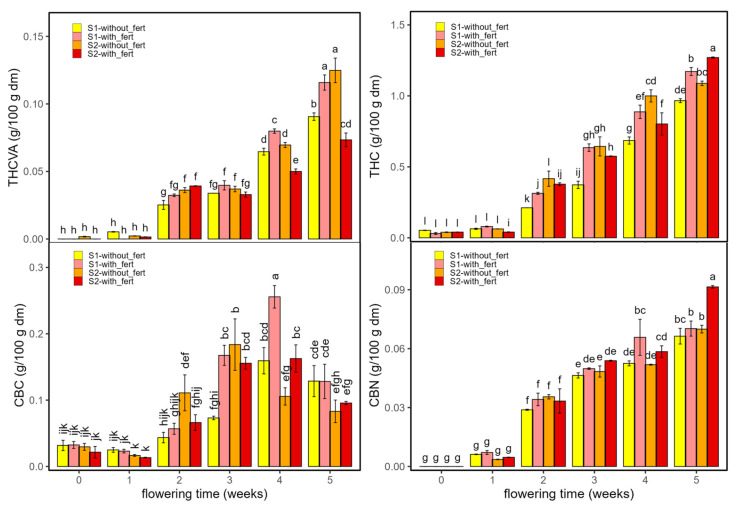
Psychoactive cannabinoids and related derivatives in *Enectaliana* cultivated with or without fertilizer, from 0 to 5 flowering weeks for two planting densities (Sector 1 (S1) ≈ 2.3 plants m^−2^; Sector 2 (S2) ≈ 4.6 plants m^−2^): THCVA, THC, CBC and CBN. Different letters indicate significant differences (*p* < 0.05).

**Figure 6:**
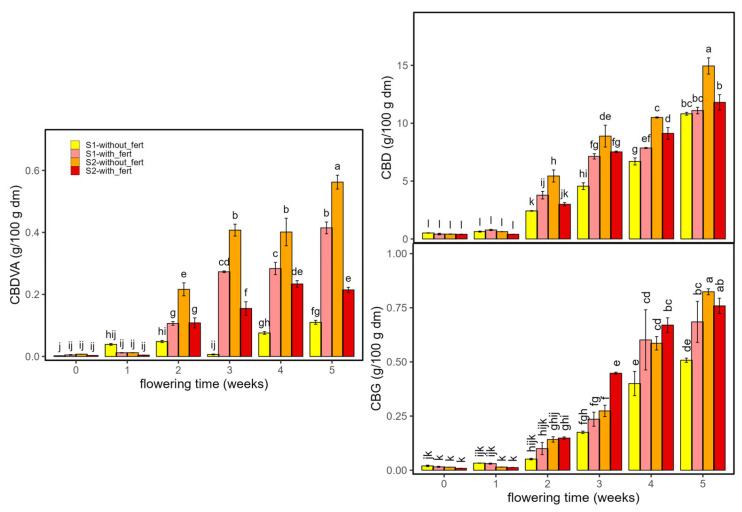
Non-psychoactive cannabinoids in *Enectaliana* cultivated with or without fertilizer, from 0 to 5 flowering weeks for two planting densities (Sector 1 (S1) ≈ 2.3 plants m^−2^; Sector 2 (S2) ≈ 4.6 plants m^−2^): CBDVA, CBD and CBG. Different letters indicate significant differences (*p* < 0.05).

**Figure 7:**
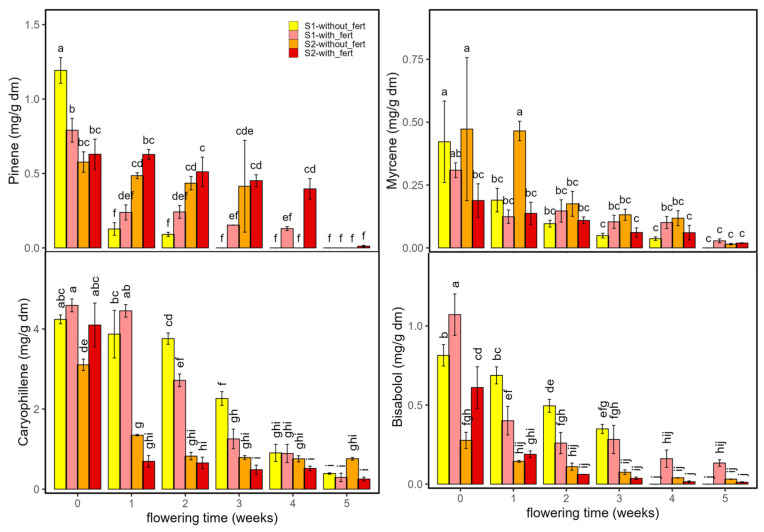
Terpene content (pinene, myrcene, caryophyllene and bisabolol mg/g dm) in *Enectaliana* cultivated with or without fertilizer, from 0 to 5 flowering weeks for two planting densities (Sector 1 (S1) ≈ 2.3 plants m^−2^; Sector 2 (S2) ≈ 4.6 plants m^−2^). Different letters indicate significant differences (*p* < 0.05).

**Figure 8:**
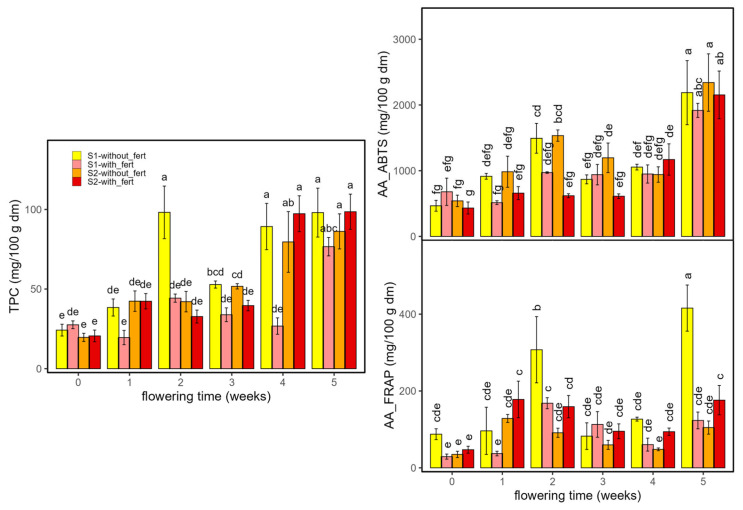
Total polyphenol content (TPC, mg GAE/100 g dm) and antioxidant activity (AA: ABTS and FRAP, mg trolox/100 g dm) in *Enectaliana* cultivated with or without fertilizer, from 0 to 5 flowering weeks for two planting densities (Sector 1 (S1) ≈ 2.3 plants m^−2^; Sector 2 (S2) ≈ 4.6 plants m^−2^). Different letters indicate significant differences (*p* < 0.05).

Below, results from a 2023 field trial in Mallorca evaluating two cultivars (*Enectaliana*, *Enectarol*) at two planting densities (Sector 1 ≈ 2.3 plants m^−2^; Sector 2 ≈ 4.6 plants m^−2^) with sampling from flowering onset (week 0) to week 5; fertilization (with/without) was additionally assessed in *Enectaliana*. In this study, “flowering time” refers to the number of weeks after floral initiation (week 0 = onset of flowering), representing the developmental progression of inflorescences rather than the calendar timing of floral onset. Treatment effects were tested by three-way ANOVA (Density × Time × Cultivar; Density × Time × Fertilization) and are reported first for density, time, and cultivar (Figure 1, Figure 2, Figure 3 and Figure 4 and key statistics in Table 1), followed by, within *Enectaliana*, density, time, and fertilization (Figure 5, Figure 6, Figure 7 and Figure 8) and key statistics in Table 2. Post hoc comparisons (Tukey’s HSD) are also shown in Figure 1, Figure 2, Figure 3, Figure 4, Figure 5, Figure 6, Figure 7 and Figure 8 as letter groupings above the bars; groups sharing a letter do not differ significantly (*p* > 0.05), whereas different letters indicate *p* < 0.05.

No significant differences were observed in moisture content across treatments (*p* > 0.05), confirming uniform drying and full compliance with the 8–13% regulatory threshold [10].

### 2.1. Effects of Cultivar, Planting Density, and Flowering Time

#### 2.1.1. Cannabinoids

Figure 1 and Figure 2 show the concentrations of psychoactive cannabinoids and their derivatives (THCVA, THC, CBC, and CBN) (Figure 1) and non-psychoactive cannabinoids (CBDVA, CBD, and CBG) (Figure 2) in the *Enectaliana* and *Enectarol* cultivars as a function of planting density and flowering time. As can be seen in these figures, across the flowering time, *Enectaliana* and *Enectarol* exhibited clearly divergent trajectories in the levels of cannabinoids.

THCVA and THC rose steadily in both sectors, with *Enectaliana* reaching much higher end-point levels than *Enectarol*. This pattern is consistent with observations by Glivar et al. [11] for the cultivars *KV Dora* and *Tisza*, where the former showed 77% higher THC than the latter. In contrast, CBC was higher in *Enectarol* from the beginning and continued to rise, whereas *Enectaliana* stayed low to moderate. Mechanistically, all major cannabinoid pathways originate from the common precursor CBGA; from this metabolite, flux is directed by specific synthases. When CBCA synthase predominates, CBGA is converted to CBCA, which upon decarboxylation yields CBC; genotypes with higher CBCA-synthase activity tend to accumulate more CBC [12]. CBN showed the sharpest cultivar divergence: it emerged in *Enectarol* at weeks 2–3 and rose steadily in both sectors but remained near zero in *Enectaliana*. Because CBN derives primarily from oxidative THC conversion, and THC was low in *Enectarol*, this pattern indicates a higher propensity for THC oxidation under our conditions. THC degradation is minimized by low temperature and slightly to moderately acidic pH during storage/processing [13].

Planting density mainly modulated magnitudes without reversing cultivar patterns: S1 tended to enhance THCVA/THC in *Enectaliana* late in flowering, while between-sector differences in *Enectarol* were modest; CBC and CBN remained higher in *Enectarol* across sectors. Overall, density acted as a scaling factor, whereas cultivar and time governed the direction and hierarchy of responses (*Enectaliana* > *Enectarol* for THCVA/THC, and *Enectarol* > *Enectaliana* for CBC/CBN).

Regarding non-psychoactive cannabinoids (Figure 2), CBDVA and CBD exhibited sustained increases in both sectors (S1 and S2) from early flowering to the end in *Enectaliana*, whereas in *Enectarol*, they remained very low or undetectable. CBG showed the opposite cultivar pattern: *Enectarol* exhibited a marked rise beginning around week 2 while *Enectaliana* stayed very low across weeks and planting densities. According to the literature, CBD and CBG typically rise as inflorescences mature, peaking around weeks 6–7, although peak timing and magnitude are genotype-dependent [14]. Genetically controlled partitioning of CBGA among CBDA/THCA/CBCA pathways varies by cultivar; in Type IV (CBG-dominant) chemotypes, reduced flux to CBDA/THCA elevates CBG, explaining *Enectarol*’s low CBD/THC and high CBG [15].

As with the psychoactive cannabinoids, planting density primarily modulated magnitude without altering cultivar patterns. In *Enectaliana*, CBDVA was higher in the lower-density sector (S1), whereas CBD was higher in the higher-density sector (S2). In *Enectarol*, S1–S2 differences were modest for CBG, with higher peaks in S2 at weeks 4–5.

Planting density can modify light, water, and nutrient use, yet its direct impact on cannabinoid biosynthesis is often limited [8]; microclimate and competition may still modulate cannabinoid levels [16,17]. In our trial, density was not a determining factor for cannabinoid production in *Enectaliana* or *Enectarol*. Overall, the literature indicates that lower densities can favour CBD/CBG in some cultivars, but effects are typically modest and genotype-dependent [18,19].

The three-way interaction (Density × Time × Cultivar) from ANOVA was significant for all compounds (*p* < 0.001) except CBN (CBN: *p* = 0.1), with moderate-to-large effect sizes for the rest (η^2^_p_ = 0.50–0.88). Across cannabinoids, the dominant two-way interaction was consistently Time × Cultivar (η^2^_p_ ≥ 0.86, often ≥ 0.95), indicating cultivar-dependent temporal trajectories. Regarding main effects, Cultivar and Time exerted similarly large influences (η^2^_p_ ≈ 0.92–1.00). Plant density showed smaller main effects but significant in CBC, CBDVA, CBN and THCVA (η^2^_p_ = 0.24–0.87). Overall, time and cultivar shaped cannabinoid accumulation; planting density mainly changed magnitudes without altering the ordering of compounds or their trajectories.

**Table 1:** Key three-way ANOVA (Density (D) × Time (T) × Cultivar (C)) outcomes by cannabinoids, terpenes and antioxidant compounds. F (F-statistic), *p* (*p*-value), and η^2^_p_ (partial eta squared; effect size).

	F (D × T×C)	*p* (D × T × C)	η^2^_p_ (D × T × C)	Two-Way Interaction	F (2-v)	*p* (2-v)	η^2^_p_ (2-v)	Main Effect	F (Main)	*p* (Main)	η^2^_p_ (Main)
THCVA	17.7	7 × 10^−10^	0.65	T × C	161	<2 × 10^−16^	0.94	T	1236	<2 × 10^−16^	0.99
THC	10.8	5 × 10^−7^	0.53	T × C	912	<2 × 10^−16^	0.99	T	1760	<2 × 10^−16^	0.99
CBC	12.2	1 × 10^−7^	0.56	T × C	60	<2 × 10^−16^	0.86	C	1453	<2 × 10^−16^	0.97
CBN	1.8	0.1	0.16	T × C	192	1.7 × 10^−30^	0.95	C	1727	2.7 × 10^−39^	0.97
CBDVA	69.8	<2 × 10^−16^	0.88	T × C	727	<2 × 10^−16^	0.99	C	5783	<2 × 10^−16^	0.99
CBD	11.6	2 × 10^−7^	0.55	T × C	1300	<2 × 10^−16^	0.99	C	10,117	<2 × 10^−16^	1
CBG	9.6	2 × 10^−6^	0.50	T × C	438	<2 × 10^−16^	0.98	C	2769	<2 × 10^−16^	0.98
α-Pinene	7.6	2.7 × 10^−5^	0.44	T × C	60.4	<2 × 10^−16^	0.86	C	1114	<2 × 10^−16^	0.96
Myrcene	11.3	3.1 × 10^−7^	0.54	T × C	24.4	3.8 × 10^−12^	0.72	T	148	<2 × 10^−16^	0.94
β-Caryophyllene	50.8	<2 × 10^−16^	0.84	T × C	95.1	<2 × 10^−16^	0.91	T	464	<2 × 10^−16^	0.98
Bisabolol	4.7	1.1 × 10^−3^	0.33	T × C	26.4	1 × 10^−12^	0.73	C	891	<2 × 10^−16^	0.95
TPC	18.4	3.7 × 10^−10^	0.66	T × C	8.61	7.0 × 10^−6^	0.47	T	149	5.3 × 10^−28^	0.94
AA (ABTS)	2	0.09	0.17	D × T	4.5	0.002	0.32	T	61	1.3 × 10^−19^	0.86
AA (FRAP)	6	0.0002	0.39	T × C	20.9	4.9 × 10^−11^	0.69	T	56	6.9 × 10^−19^	0.85

#### 2.1.2. Terpenes

Figure 3 shows the evolution of α-pinene, myrcene, β-caryophyllene, and bisabolol across the two cultivars, *Enectaliana* and *Enectarol*, under the two planting densities. D-limonene results are not shown as concentrations were ≤0.08 mg/g dm under all conditions. As can be seen in Figure 3, the four terpenes peaked at flowering onset (week 0) and declined along the weeks.

At week 0, α-pinene in *Enectaliana* was 0.79 ± 0.08 mg/g dm (S1) and 0.63 ± 0.10 mg/g dm (S2), then declined over time, most markedly in S1. In *Enectarol*, initial α-pinene was 0.34 ± 04 mg/g in S1 and ≤0.01 mg/g dm in S2. By week 5, α-pinene was nearly negligible in both cultivars and sectors. This pattern aligns with reports that higher-THC genotypes show higher α-pinene [3] and that pinene declines during flowering [20]. Also, a similar response to planting density for α-pinene was reported by Mirjalili et al. [21] in summer savoury, where α-pinene increased with plant density.

Myrcene also peaked at the onset of flowering with the highest value in *Enectarol*-S2. Thereafter, it dropped sharply and, from week 1 onward, remained low (≤~0.15 mg/g dm) approaching zero by weeks 4–5. No systematic density effect or reversal of cultivar patterns was observed. This behaviour aligns with reports linking higher-THC genotypes to higher myrcene [3], the lack of sector effects in comparable cultivars [22], and the general decline of myrcene after early flowering [23].

β-caryophyllene was consistently higher in *Enectaliana* than in *Enectarol*, with the largest cultivar gaps at early time points; values then converged to low levels by weeks 4–5. Bisabolol exhibited the opposite hierarchy, being much higher in *Enectarol* (still elevated through weeks), while *Enectaliana* remained low, underscoring the importance of genetic background in terpene content [24]. Planting density mainly modulated magnitudes at early stages without reversing cultivar rankings; within-week differences are indicated by Tukey’s HSD letter groupings in Figure 3, indicating that planting density had no significant effect on bisabolol (*p* > 0.05). These trends are broadly consistent with prior reports (e.g., Felina 32 vs. Uso 31) showing early maxima and subsequent declines [1,25]. Note that the literature also reports mixed cultivar effects for bisabolol (e.g., no significant differences in some comparisons) and limited density sensitivity in related species [26].

The three-way ANOVA (Table 1) revealed significant three-way interactions for all compounds (η^2^_p_ ≈ 0.33–0.84). Among two-way interactions, Time × Cultivar consistently predominated across terpenes, indicating genotype-dependent temporal trajectories (η^2^_p_ ≈ 0.72–0.91). Regarding main effects, Time emerged as the leading determinant for myrcene and β-caryophyllene (η^2^_p_ ≈ 0.94–0.98), whereas Cultivar dominated α-pinene and bisabolol (η^2^_p_ ≈ 0.95–0.96). Density acted primarily as a modulator, showing clear main effects for α-pinene, β-caryophyllene and bisabolol (η^2^_p_ ≈ 0.46–0.86), but little to no main effect on myrcene (*p* > 0.05). Collectively, these results indicate that temporal dynamics shaped by genotype govern terpene evolution, while planting density chiefly scales magnitudes via interactions rather than overturning the overall temporal hierarchy.

Differences between *Enectaliana* and *Enectarol* in cannabinoid and terpene profiles were consistent with their genetic specialization towards CBD- and CBG-type chemotypes, respectively. The marked variation observed along the flowering progression reflects the dynamic regulation of secondary metabolism, which is tightly linked to floral development and environmental cues such as temperature and radiation intensity. Similar cultivar-dependent trends have been reported by Andre et al. [27] and Calzolari et al. [26], who demonstrated that genotype is the primary determinant of phytochemical composition, with phenological stage acting as a secondary modulator. In our trial, the accumulation peaks observed during mid-flowering suggest a coordination between trichome maturation and the biosynthesis of both cannabinoids and volatile terpenes, in line with recent metabolomic evidence showing that CBG and monoterpenes share early biosynthetic precursors derived from the plastidial MEP pathway [28].

#### 2.1.3. Antioxidant Compounds

Figure 4 shows the results of Total polyphenol content (TPC, mg gallic acid/100 g dm) and antioxidant activity (AA, ABTS and FRAP assays, mg trolox/100 g dm) of hemp samples according to cultivar, planting density, and flowering time.

Total phenolics (TPC) were low and fairly stable through weeks 0–3 (mean 32.8 ± 10.7 mg/100 g dm), then rose sharply at weeks 4–5 across cultivars and densities. At week 4, TPC was higher in *Enectarol*, whereas *Enectaliana*–S2 lagged. By week 5, groups converged to high values (mean of 103.5 ± 12.0 mg/100 g dm), except for *Enectaliana*–S2 ending at 76.6 ± 5.8 mg/100 g dm. Within-week differences are indicated by Tukey’s HSD letter groupings in Figure 4.

Antioxidant activity rose with flowering, with a marked late surge. The AA according to ABTS assay increased modestly through weeks 0–4 (mean of 833 ± 313 mg/100 g dm) and then jumped at week 5 across groups; the highest values occurred in *Enectarol*, particularly at the higher density (S2) (3292 ± 1263 mg/100 g dm), while *Enectaliana* remained lower after 5 weeks in both sectors (mean 2036 ± 272 mg/100 g dm). The AA according to FRAP assay was more variable early, but likewise showed a pronounced peak at week 5, dominated by *Enectarol*–S2. Planting density chiefly scaled magnitudes at late stages (no reversals of cultivar ranking), with S2 ≥ S1 at late peaks, compatible with studies showing that developmental stage and canopy microclimate shape phenolic composition and reducing capacity in aromatic species [29]. Within-week differences are indicated by Tukey’s HSD letter groupings in Figure 5. Overall, these antioxidant trends mirror the late-flowering rise in phenolics and agrees with findings in aromatic/medicinal species that peak at full bloom or near maximal flowering and are strongly phenology-dependent (e.g., *Salvia officinalis* [30] and *Origanum vulgare* [29]).

The three-way ANOVA (Table 1) revealed significant three-way interactions for TPC (η^2^_p_ = 0.66) and FRAP (η^2^_p_ = 0.18); ABTS did not show a significant three-way term (*p* > 0.05). Among two-way interactions, Time × Cultivar was the most influential for TPC (η^2^_p_ = 0.47) and FRAP (η^2^_p_ = 0.69), revealing genotype-dependent temporal trajectories, whereas ABTS was chiefly modulated by Density × Time (η^2^_p_ = 0.32), consistent with a density-driven microclimate effect on antioxidant activity. Across variables, Time emerged as the dominant main effect (η^2^_p_ = 0.85–0.94), indicating that phenolic content and antioxidant activity are primarily governed by flowering progression. In contrast, Cultivar and Density acted mainly as modulators expressed through two- and three-way interactions. Taken together, these patterns show that temporal dynamics set the overall trajectories, while cultivar shapes their timing and amplitude, and planting density primarily scales magnitudes without overturning the temporal hierarchy.

### 2.2. Effect of Fertilizer Application, Planting Density, and Flowering Time in the Enectaliana Cultivar

#### 2.2.1. Cannabinoids

Figure 5 and Figure 6 show the concentrations of psychoactive (THCVA, THC, CBC, and CBN) (Figure 5) and non-psychoactive (CBDVA, CBD, and CBG) (Figure 6) cannabinoids for samples grown with and without fertilizer as a function of planting density and flowering time. THC was the most abundant psychoactive cannabinoid under all conditions.

THCVA and THC were stable from week 0 to 1, then rose progressively from weeks 2 to 5 across treatments. Fertilizer effects emerged mainly at late flowering: in S1, week-5 levels were higher in fertilized plots (THC by 30–50%, THCVA by 30–45%); in S2, fertilizer modestly increased THC (15–25%), whereas THCVA was 10–20% higher in unfertilized plots. These late-rising THC/THCVA trajectories are consistent with reports of progressive cannabinoid accumulation modulated by genotype and environment [31]. Overall, fertilization and density scaled magnitudes but did not alter the temporal course.

CBC increased from near-baseline at weeks 0–1 to maxima at weeks 3–4, then declined by week 5. Treatment differences (Fertilizer × Density) were modest and week-specific, without reversals of ranking (see Tukey letters in Figure 6). CBN emerged at weeks 1–2 and rose steadily to week 5, with fertilized plots tending to show the highest values, consistent with greater oxidative conversion of THC to CBN under advanced maturity [13]; density mainly scaled magnitudes. Overall, fertilization and density modulated amplitude, whereas flowering time dictated the trajectories of CBC (rise–peak–decline) and CBN (monotonic increase). Consistent with nutrient-response literature, fertilization effects can be non-linear with intermediate N and P optima: increasing supply may raise THC/THCVA at certain doses but not necessarily CBC/CBN, and excessive N can depress cannabinoids [32,33].

Similarly, CBDVA, CBD and CBG showed steady contents for weeks 0 and 1 and increased with flowering, with the strong rises from week 2 to 5 under both densities and nutrient regimes. CBDVA rose from near-baseline to mid/late-flowering maxima, with the highest end values typically in S2 without-fertilizer. CBD increased steadily across weeks, reaching the greatest levels at week 5, again highest in S2 without-fertilizer and next in S2 with-fertilizer. CBG remained low until ~week 2–3, then climbed to late-flowering peaks, with S2 without-fertilizer generally leading. Fertilizer and density effects were modest and week-specific, chiefly scaling magnitudes at late stages; the Tukey letter groupings in Figure 6 indicate the within-week differences. From a management perspective, the fertilizer responses observed here align with the concept of non-linear nutrient–response curves and intermediate N–P optima during flowering: increasing nutrient supply can enhance cannabinoid accumulation within a favourable window, yet excess nitrogen may depress CBDA/THCA, which helps explain the end-of-cycle advantage of plants grown without fertilizer in S2 for CBDA, while S1 benefits from fertilization [32,33].

The three-way interaction (Density × Time × Fertilizer) from ANOVA (Table 2) was significant for all cannabinoids (*p* < 0.001) with moderate-to-large effect sizes (η^2^_p_ ≈ 0.42–0.97). Among two-way interactions, Density × Fertilizer predominated for THCVA, CBDVA, and CBD (η^2^_p_ *>* 0.81); Time × Fertilizer for THC (η^2^_p_ ≈ 0.88); and Density × Time for CBC, CBG, and CBN (η^2^_p_ *>* 0.57). Across variables, Time emerged as the dominant main effect (η^2^_p_ ≥ 0.99) and fertilization effects were important in CBC, CBD, CBG, CBN, THC and THCVA (η^2^_p_ ≈ 0.18–0.54). Taken together, fertilizer chiefly modulated amplitude, often most evident at late flowering and varying by density, without consistently reordering the hierarchy among compounds or altering their temporal trajectories previously described.

**Table 2:** Key three-way ANOVA (Density (D) × Time (T) × Fertilizer (f)) outcomes by cannabinoids, terpenes and antioxidant compounds. F (F-statistic), *p* (*p*-value), and η^2^_p_ (partial eta squared; effect size).

	F (D × T × f)	*p* (D × T × f)	η^2^_p_ (D × T × f)	Two-Way Interaction	F (2-v)	*p* (2-v)	η^2^_p_ (2-v)	Main Effect	F (Main)	*p* (Main)	η^2^_p_ (Main)
THCVA	85	<2 × 10^−16^	0.90	D × f	227	<2 × 10^−16^	0.83	T	2160	<2 × 10^−16^	1
THC	57	<2 × 10^−16^	0.90	T × f	73	<2 × 10^−16^	0.88	T	2485	<2 × 10^−16^	1
CBC	10	1 × 10^−6^	0.59	D × T	24	6 × 10^−12^	0.71	T	776	<2 × 10^−16^	0.99
CBN	8	2 × 10^−5^	0.49	D × T	13	6 × 10^−8^	0.57	T	1311	<2 × 10^−16^	0.99
CBDVA	272	<2 × 10^−16^	0.90	D × f	1715	<2 × 10^−16^	0.97	T	954	<2 × 10^−16^	0.99
CBD	20	1 × 10^−10^	0.70	D × f	208	<2 × 10^−16^	0.81	T	2220	<2 × 10^−16^	1
CBG	7	6 × 10^−5^	0.40	D × T	14	1 × 10^−8^	0.60	T	635	<2 × 10^−16^	0.98
α-Pinene	5.8	3 × 10^−4^	0.4	D × T	41	3 × 10^−16^	0.8	T	142	<2 × 10^−16^	0.94
Myrcene	10.3	10 × 10^−7^	0.5	D × T	21	4 × 10^−11^	0.7	T	65	<2 × 10^−16^	0.87
β-Caryophyllene	11.5	2 × 10^−7^	0.5	D × T	113	<2 × 10^−16^	0.9	T	482	<2 × 10^−16^	0.98
Bisabolol	9.8	2 × 10^−6^	0.5	D × T	32	3 × 10^−14^	0.8	T	233	<2 × 10^−16^	0.96
TPC	38.7	1 × 10^−15^	0.8	D × T	129	<2 × 10^−16^	0.93	T	745	<2 × 10^−16^	0.99
AA (ABTS)	12.7	7.3 × 10^−8^	0.57	T × f	69	<2 × 10^−16^	0.88	T	869	<2 × 10^−16^	0.99
AA (FRAP)	78.8	<2 × 10^−16^	0.89	D × f	616	<2 × 10^−16^	0.93	T	256	<2 × 10^−16^	0.96

#### 2.2.2. Terpenes

Figure 7 shows the evolution of α-pinene, myrcene, β-caryophyllene and bisabolol across the two planting densities (S1, S2) and two fertilization regimes (with and without fertilizer). D-limonene results are not shown as concentrations were ≤0.08 mg/g dm under all conditions. All four terpenes peaked at flowering onset (week 0) and declined thereafter.

For α-pinene and myrcene, fertilized plots were generally higher than unfertilized within the same week, most noticeably at early to mid stages, while late-flowering values converged toward low levels. β-Caryophyllene and bisabolol were the predominant terpenes at the start of flowering; β-caryophyllene was greatest at week 0–1, particularly in fertilized treatments, and bisabolol likewise showed early maxima accentuated by fertilization. Planting density modulated amplitudes: the low-density sector (S1) tended to show higher α-pinene and myrcene, especially early, whereas the high-density sector (S2) favoured β-caryophyllene and bisabolol at weeks 0–1. Fertilization increased levels within a given density without altering trajectories or reversing the S1/S2 hierarchy. By late flowering, all treatments converged to low values. Week-specific differences among the four treatment combinations (S1/S2 × with/without fertilizer) are indicated by Tukey’s letter groupings. Overall, fertilization modesty and episodically increased terpene levels modestly and episodically, acting as an amplitude modulator rather than a driver of temporal pattern. These treatment effects fit the emerging agronomic literature: nitrogen supply and form can alter terpene biosynthesis in cannabis in a compound-specific, non-linear manner: monoterpenes (such as α-pinene and myrcene) often decrease as N increases, sesquiterpenes (like β-Caryophyllene and bisabolol) are less sensitive at low N but can decline at excessive N, and high NH_4_^+^:NO_3_^−^ ratios depress both terpenes and yield [33].

Key statistics of the three-way ANOVA are shown in Table 2. Flowering time was the dominant main effect for all terpenes (η^2^_p_ ≈ 0.87–0.98). Density had large main effects for the β-caryophyllene (η^2^_p_ = 0.93) and bisabolol (η^2^_p_ = 0.89), but moderate for α-pinene (η^2^_p_ = 0.51) and negligible for myrcene. Fertilizer had smaller main effects, significant for α-pinene, myrcene and β-caryophyllene (η^2^_p_ ≈ 0.18–0.24), but not for bisabolol. Interactions were substantial and trait-specific: Density × Time was strong for all four (η^2^_p_ ≈ 0.69–0.92), Density × Fertilizer was notable for myrcene (η^2^_p_ = 0.30) and weaker/absent for the others, and Time × Fertilizer was consistently important (η^2^_p_ = 0.52–0.73). The three-way Density × Time × Fertilizer term was significant for all compounds (η^2^_p_ = 0.38–0.55, indicating context-dependent fertilizer responses. Overall, time governs the trajectories, density strongly shapes sesquiterpene amplitudes, and fertilization mainly modulates magnitudes via interactions rather than as a standalone driver.

#### 2.2.3. Antioxidant Compounds

Figure 8 presents the results of the TPC and antioxidant activity assessed by ABTS (AA-ABTS), and FRAP (AA-FRAP) for samples grown with and without fertilizer, at two planting densities (S1 and S2) and across multiple flowering times. TPC and antioxidant activity rose with flowering, with the sharpest increases at weeks 4–5. Fertilization generally elevated values within a given density, most visibly at late stages; density mainly scaled magnitudes (treatment order preserved). In TPC, fertilized S1 often led at peak weeks, while in AA_ABTS late maxima were higher in S2 treatments. AA_FRAP was more variable early but showed a pronounced week-5 surge, again strongest under fertilization (notably S1 with fertilizer). Week-specific differences among the four combinations (S1/S2 × with/without fertilizer) are denoted by Tukey letter groupings.

These patterns are consistent with the three-way ANOVA (Table 2): time was the dominant main effect for TPC and AA (η^2^_p_ = 0.96–0.99) and fertilization exerted strong to substantial main effects (η^2^_p_ = 0.73–0.89). Density showed a moderate main effect on TPC and AA_ABTS (η^2^_p_ = 0.47–0.48), but was very strong for AA_FRAP (η^2^_p_ = 0.74). Interactions were pronounced: Density × Time ranged η^2^_p_ ≈ 0.60–0.93 (lowest in AA_ABTS, highest in TPC); Density × Fertilizer showed the widest span (0.28–0.93, from modest in AA_ABTS to very large in AA_FRAP, with TPC at 0.73); Time × Fertilizer was consistently large across traits (0.80–0.88); and the three-way Density × Time × Fertilizer term remained substantial (0.57–0.89). Collectively, these ranges indicate robust context dependence, with fertilizer effects amplified by both developmental stage and stand density. Although many hemp studies focus on seeds or co-products, there is broad agreement that ABTS/FRAP track phenolic load and its variation with management and genotype [34].

The progressive increase in total phenolic content (TPC) and antioxidant activity (ABTS, FRAP) towards late flowering suggests an adaptive metabolic response to reproductive and oxidative stress. Phenolic biosynthesis in *Cannabis sativa* has been shown to intensify under conditions of high irradiance and limited nitrogen availability, typical of organic cropping systems [35,36]. The slightly higher antioxidant capacity in Eckosil-treated plants supports the hypothesis that silicon supplementation enhances the phenylpropanoid pathway and the activity of ROS-scavenging enzymes, as previously observed in basil and sage [37,38]. Such responses underline the multifunctional role of Si as a biostimulant improving secondary metabolism and stress resilience, consistent with the objectives of sustainable hemp production under Mediterranean conditions.

Overall, the patterns observed across cultivars and fertilization regimes highlight a common metabolic trajectory during floral development, characterized by early accumulation of cannabinoids and terpenes followed by a late increase in phenolic antioxidants. This coordinated progression reflects a shift from primary biosynthetic investment to stress-protective metabolism, modulated by genotype and nutritional status. Such dynamics are consistent with reports in other aromatic crops where the balance between carbon allocation and oxidative pressure determines the final phytochemical profile [38,39,40].

## 3. Materials and Methods

### 3.1. Experimental Site

This study was carried out in the field of the company NOMA (Balearic Islands Hemp Cooperative) located in the town of Binissalem (Balearic Islands, Spain) during 2023. The two soil sectors used for hemp cultivation initially exhibited similar characteristics. The soil was classified as clay-loam with a pH of 8.1 ± 0.1 and organic matter ranging 2.7–4.2%. Available nutrients were as follows: phosphorus = 19 mg/kg, potassium = 55 mg/kg, calcium = 16.24 meq/100 g, magnesium = 0.44 meq/100 g, and sodium = 128 mg/kg. The soil was highly calcareous and rich in CaCO_3_, with normal to high Mg and Na contents. According to Adesina et al. [41], the described soil meets the optimal conditions for hemp cultivation.

**Figure 9:**
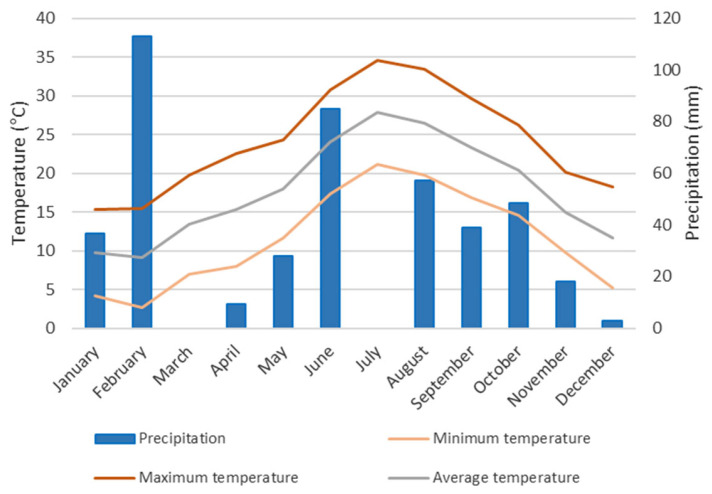
Climograph of data from the Binissalem meteorological station (2023).

Figure 9 presents the climograph based on 2023 data from the Binissalem meteorological station (Mallorca, Spain). Mean monthly temperature ranged from 9.3 °C to 26.8 °C. The maximum temperature recorded was 34.4 °C in July, and the minimum was 3.7 °C in January. Mean monthly precipitation ranged from 0 mm in March and June to 113 mm in February.

### 3.2. Samples

Two cultivars of hemp were used in this study: *Enectaliana*, which is rich in cannabidiol (CBD), and *Enectarol*, which is rich in cannabigerol (CBG). Both cultivars are registered industrial monoecious varieties of *Cannabis sativa* L., authorized in the European Union Common Catalogue, with THC content below the legal limit of 0.3% (*w*/*w*). The samples were cultivated in Mallorca using traditional farming methods provided by the “*Cooperativa de Cáñamo de las Islas Baleares (NOMA)*”.

The experimental field was divided into two sectors with different plant densities: Sector 1 (S1): 2.3 plants/m^2^ and Sector 2 (S2): 4.6 plants/m^2^. Within each sector, three randomized plots per cultivar (and per fertilization treatment in *Enectaliana*) were established to provide replication. Each plot included five plants randomly selected for sampling at each flowering week. Although planting density was applied at the sector level, replication was maintained within each sector at the plot level to allow valid statistical inference. Accordingly, each sector contained replicated experimental plots for every combination of cultivar (and fertilization treatment in *Enectaliana*) and sampling week. Plot means were used as the experimental units for statistical analysis, providing an appropriate estimate of within-sector variance for each treatment combination. In both sectors, plots were fertilizer with Eckosil, as silicon-based biostimulant containing 1.2% orthosilicic acid (Si(OH)_4_) and trace micronutrients (Zn 0.05%, Mn 0.05%, Fe 0.1%) used to enhance plant vigour. The crop was managed under organic farming conditions, and therefore no additional N, P, or K fertilizers were applied during the trial to isolate the effects of this formulation on plant metabolic responses. Additionally, the *Enectaliana* cultivar was cultivated with or without fertilizer, respectively, to evaluate the effect of Eckosil application. In each sector, the cultivars *Enectaliana* and *Enectarol* were arranged in alternating randomized plots. Within the *Enectaliana* cultivar, fertilized and unfertilized treatments were also randomly assigned within each block. Each plot measured approximately 4 m × 3 m and contained enough plants to allow random sampling of five plants per plot at each flowering week. This layout provided three independent replicates for every treatment combination and minimized positional effects. The sector factor (S1 vs. S2) was treated as a fixed effect in the statistical models, while replication within sectors (plots and blocks) accounted for local variability and served as the source of residual variance.

The experimental layout followed a randomized complete block design with three independent replicates per treatment. Each block contained all treatment combinations (cultivar × density, and in *Enectaliana* also × fertilization), randomly assigned to individual plots within the corresponding sector to minimize soil and microclimatic gradients.

From the onset of flowering on 21 August 2023, until its conclusion on 1 October 2023, weekly samplings were conducted for subsequent analysis. The analyzed samples consisted of inflorescences (flowers) harvested from *Cannabis* plants. Sample collection followed the procedures outlined in Regulation (CE) No 1177/2000 [10]. For each condition, three samples were collected. After harvesting, stems and seeds larger than 2 mm were removed, the remaining material was dried at 70 °C until constant weight, and ground and sieved to obtain a powder with a particle size of <0.1 mm. The samples were stored in a dark place at a temperature below 25 °C until further analysis (less than 3 days).

### 3.3. Analytical Methodology

#### 3.3.1. Moisture Content

The experimental determination of the moisture content in hemp samples was conducted following the method described by the European Commission for the quantitative determination of Δ^9^-THC in hemp [10]. Approximately 200 mg of the sample was weighed and dried in an oven at 90 °C until constant weight.

#### 3.3.2. Extraction of Bioactive Compounds

Bioactive compounds extraction was performed following the methodology described by De Prato et al. [42] with some modifications. Approximately 100 mg of the powdered samples were weighed, and 10 mL of methanol was added. Extraction was carried out in an ultrasonic bath for 15 min, followed by centrifugation at 3000 rpm for 10 min (Unicen 21, Ortoalresa, Madrid, España). The supernatant was filtered through a 0.45 µm pore size membrane filter and stored in a freezer at −80 °C until further analysis.

#### 3.3.3. Cannabinoid Analysis by GC-FID

The content of various cannabinoids (CBDV, THCV, CBC, THC, CBD, CBN, CBG) in the methanolic extracts was analyzed following the method established by the European Commission for the quantitative determination of THC content in hemp [10], with slight modifications according to Callado et al. [2] for the simultaneous determination of the cannabinoids. An Agilent HP 5890 GC gas chromatograph (Hewlett-Packard, Wilmington, DE, USA) equipped with a flame ionization detector (FID) and an autosampler (7683B Agilent Technologies, Barcelona, Spain) was used. The column used was an SC-5 MS (Scharlab, Barcelona, Spain) 30 m length, 0.25 mm internal diameter, and 0.25 µm thickness, with a stationary phase of 5% phenyl-95% methylpolysiloxane. Helium was used as the carrier gas at a constant flow rate of 1.3 mL/min. The inlet temperature was 300 °C without a split. The injection volume was 1 µL. The oven temperature was programmed starting at 160 °C (held for 2 min), ramped to 242 °C at 25 °C/min, then to 250 °C at 2 °C/min, to 300 °C at 25 °C/min and finally held at 305 °C for 5 min.

For cannabinoid quantification, calibration curves were prepared for each cannabinoid. The calibration curve for CBDV and THCV consisted of solutions with concentrations of 10–50 ppm. For CBD, CBG, and CBN, the concentrations were 30, 60, 90, 120, and 150 ppm. For CBC and THC, the concentrations were 20–100 ppm. Cannabinoid concentrations were expressed in g/100 g dry matter (dm).

The reported values correspond to neutral cannabinoids quantified in a concentrated dry extract, expressed on a dry-extract basis. Therefore, it is not directly comparable to the in-plant THC percentage used for agronomic/legal compliance (≤0.3% THC in EU-listed industrial hemp).

#### 3.3.4. Terpene Analysis by GC-MS

The quantification of some terpenes in the hemp samples was performed following the method described by Ibrahim et al. [3]. Analyses were performed using a gas chromatograph (Agilent 6890A, Agilent Technologies, Wilmington, DE, USA) equipped with an Agilent 5975 mass spectrometer detector and an Agilent 7683B autosampler (Agilent 6890A, Agilent Technologies, Wilmington, DE, USA). The column used was an SC-5 MS capillary column (30 m × 0.25 mm I.D., 0.25 µm film thickness; Scharlab, Barcelona, Spain). Helium was used as the carrier gas at a constant flow rate of 1 mL/min. The inlet temperature was 250 °C with a split ratio of 15:1. The injection volume was 1 µL. The oven temperature was programmed starting at 50 °C (held for 2 min), ramped to 85 °C at 2 °C/min, then to 165 °C at 3 °C/min, and finally held at 165 °C for 10 min. The mass spectrometer was operated in full-scan mode from 40 to 450 *m*/*z*. The ionization energy was 70 eV. The ion source temperature was 230 °C and the quadrupole temperature was 150 °C. A solvent delay of 4 min was applied. The transfer line temperature was 280 °C. Compound identification was performed using the NIST14 software (National Institute of Standards and Technology, distributed by Agilent Technologies).

Standard solutions of each terpene (α-pinene, myrcene, D-limonene, β-caryophyllene, and bisabolol) were prepared in methanol. The terpene standards were mixed and adjusted to a concentration of 200 µg/mL, from which different dilutions were prepared to individual calibration curve points. n-Tridecane (C_13_ hydrocarbon) was used as the internal standard. A stock solution with a concentration of 200 µg/mL in methanol was prepared and added to all calibration solutions and sample solutions. Twelve calibration points ranging from 0.75 to 200 µg/mL were prepared from the stock solution of terpene standards and the internal standard (0.75–200 µg/mL). The internal standard concentration in each calibration curve point was 30 µg/mL. These solutions were used to construct individual terpene curves. Samples were prepared from methanolic extracts. To 2 mL of extract, the internal standard was added and treated with 1.5 mg of activated charcoal, then centrifuged at 4200 rpm for 5 min. The supernatant was filtered through a 0.45 µm pore size membrane filter and stored in a freezer at −80 °C until further analysis. Results were expressed as mg of terpene/g of dry matter (dm).

#### 3.3.5. Antioxidant Compounds

The TPC was determined using the Folin–Ciocalteu assay as described by Raposo et al. [43] with several modifications adapted for microplate analysis [44]. Microplates with 96 wells (12 columns and 8 rows) were used. For the measurement, 95 µL of H_2_O was placed in each well, adding 5 µL of Folin–Ciocalteu reagent, and 10 µL of methanolic extract. The microplate was incubated at 25 °C for 5 min, and then 80 µL of 7.5% (*w*/*v*) Na_2_CO_3_ was added to each row to stop the reaction. Then, absorbance was measured at 765 nm every 5 min for 30 min, using a UV-Vis-NIR spectrophotometer (MultiSkan spectrum, Thermo Scientific, Waltham, Massachusetts, EEUU). Total polyphenol content was determined using a calibration curve of gallic acid ranging from 25 to 250 ppm. Results were expressed as mg gallic acid (GAE)/100 g of dry matter (dm).

Antioxidant activity (AA) attributed to a wide variety of compounds in the plant involves multiple mechanisms of action from a chemical perspective. Therefore, no single analytical method can quantify antioxidant activity alone, and a combination of different methods is recommended [45]. In this study, two spectrophotometric assays were considered: ABTS and FRAP, based on electron transfer reactions. The ABTS (2,2′-azino-bis(3-ethylbenzothiazoline-6-sulfonic acid)) assay is based on the ability of antioxidant compounds in the sample to neutralize the ABTS^+^ radical cation, which is blue-green [46]. The ABTS assay was adapted for microplate analysis with some modifications [47]. ABTS reagent was prepared by mixing 1:1 K_2_S_2_O_8_ (7.5 mM) and a 2.6 mM ABTS solution (1:1). The mixture was left to react in the dark for 16 h. Subsequently, 8 mL of the prepared ABTS solution was mixed with 100 mL of EtOH (25:75). In each well of the microplate, 190 µL of ABTS reagent was added and incubated at 25 °C for 10 min. After this time, absorbance (Ao) was measured at 734 nm, then 10 µL of sample was added to each well to initiate the reaction. The mixture was left to react under the same conditions for 20 min, after which absorbance was measured at the same wavelength (A1). The antioxidant activity was calculated from the difference between A1 and Ao, correlated with a calibration curve obtained from known concentrations (25 to 800 µM) of the Trolox standard. Results were expressed as mg Trolox/100 g of dry matter (dm). The FRAP (Ferric Reducing Antioxidant Power) assay is based on the ability of antioxidant compounds in the sample to reduce ferric ion (Fe^3+^) to ferrous ion (Fe^2+^), measured by the formation of a coloured ferrous-tripyridyltriazine complex [48]. The FRAP assay was adapted for microplate analysis with some modifications [47]. To prepare the FRAP reagent on the day of analysis, three different solutions were mixed in the proportion (1:1:10, *v*/*v*/*v*): a 0.01 M TPTZ (2,4,6-tripyridyl-s-triazine) solution, a 0.02 M FeCl_3_·6H_2_O solution, and an acetic-acetate buffer (pH 3.6). The termination and calculation procedures were the same as described for ABTS assay, except the readings were taken at 593 nm. AA results were expressed as mg Trolox/100 g of dry matter (dm).

### 3.4. Statistical Analysis

All samples were analyzed in triplicate, and the results are presented as mean ± standard deviation. Two separate three-way analyses of variance (ANOVA) followed by Tukey’s Honestly Significant Difference (HSD) test were performed. The first ANOVA was conducted to evaluate the combined influence of cultivar, planting density, and flowering time on the quantitative variables of interest across all samples. The second ANOVA focused exclusively on the *Enectaliana* cultivar to assess the effects of fertilizer application, planting density, and flowering time. The analyses were performed using the mean values obtained from each experimental plot described in Section 3.2, which served as the experimental units within the randomized complete block design. This structure provided three independent replicates per treatment combination, allowing valid estimation of residual variance. All statistical analyses were conducted in R software (version 4.5) using RStudio IDE and the *agricolae* and *multcomp* packages. Model assumptions of normality and homogeneity of variances were verified using Shapiro–Wilk and Levene’s tests, respectively, before conducting the analyses. For factors showing significant main effects (*p* < 0.05), pairwise comparisons were conducted using Tukey’s HSD test (R 4.5, *agricolae* and *multcomp* packages, with RStudio IDE). When interaction effects were significant (*p* < 0.05), post hoc analyses were applied to the corresponding interaction terms using the same procedure. Planting density (sector S1 vs. S2) was included as a fixed factor, implemented at the sector level with replicated plots for each treatment combination within sectors. Therefore, density effects were interpreted as contextual (sector-level) contrasts supported by within-sector replication, whereas main inferences were drawn from the replicated cultivar, fertilization, and flowering-time effects.

## 4. Conclusions

This study shows that cultivar and harvest timing (flowering stage) are the primary determinants of metabolite accumulation in *Cannabis sativa* L., whereas planting density and fertilization act as second-order modulators that adjust concentrations without altering genotype hierarchies. Practically, cultivar choice and harvest scheduling are the main levers to maximize desired cannabinoids, optimize terpene profiles, and enhance antioxidant activity, while maintaining low THC throughout the evaluation period.

Across the flowering cycle, the two genotypes displayed distinct and stable cannabinoid signatures: *Enectaliana* consistently showed a CBD/CBDVA-dominant profile, whereas *Enectarol* was CBG-predominant, indicating robust genetic differentiation that persisted over time. Flowering time accounted for most of the variance in concentrations, yielding well-defined temporal trajectories while THC remained uniformly low under all conditions. Planting density primarily scaled the magnitude of responses without altering the rank order between genotypes. In *Enectaliana*, fertilization effects were non-linear, suggesting that nutrient supply should be calibrated to specific objectives, such as enhancing CBD accumulation without compromising other metabolites.

Terpene accumulation was characterized by early maxima followed by progressive declines over the flowering period, with the precise trajectories shaped by genotype. Planting density and fertilization played subsidiary roles. Practically, to maximize aroma and sensory quality, prioritize early, cultivar-specific harvest windows. Total phenolics and antioxidant activity rose steadily through time, consistent with functional maturation alongside flowering, supporting later harvest when the objective is to maximize antioxidant functionality.

From an agronomic standpoint, planting density operates as a fine-tuning lever that adjusts metabolite concentrations without overriding the dominant influences of cultivar and harvest timing; this makes it useful for locally balancing yield against concentration targets. Fertilization should be applied selectively and with clear objectives, since its effects are contingent on the specific cultivar–density–time combination. An integrated management sequence that begins with cultivar choice, proceeds to a cultivar-specific harvest window, and then calibrates density and fertilization enables precise targeting of outcomes such as CBD-/CBG-rich extracts, tailored terpene profiles, or enhanced antioxidant activity, all while maintaining regulatory compliance through sustained control of THC.

Looking ahead, these patterns should be validated across multiple seasons and edaphoclimatic contexts. Modelling cultivar-specific kinetics will help define optimal harvest windows for each attribute of interest (cannabinoids, terpenes, TPC/ABTS/FRAP) and align them with industrial goals including extraction efficiency, product stability, and sensory quality. Coupling these trajectories with techno-economic and environmental assessments will yield more robust, scalable management recommendations across the value chain.

## Data Availability

The data supporting the findings of this study are subject to contractual confidentiality with the industrial provider of the samples and cannot be publicly shared. De-identified/aggregated data relevant to the conclusions are included in the article. Additional information may be available from the corresponding author on reasonable request, subject to a nondisclosure agreement.

## Abbreviations

The following abbreviations are used in this manuscript:

AA	Antioxidant activity (mg trolox/100 d dry matter)
ABTS	2,2′-azino-bis(3-ethylbenzothiazoline-6-sulfonic acid)
CBC	Cannabichromene
CBD	Cannabigerol
CBDV	Cannabidivarin
CBG	Cannabigerol
CBN	Cannabinol
FRAP	Ferric Reducing Antioxidant Power
GC-FID	Gas Chromatography with Flame ionization detector
CG-MS	Gas Chromatography with Mass Spectrometry
THC	Tetrahydrocannabinol
THCV	Tetrahydrocannabivarin
TPC	Total polyphenol content (mg gallic acid/100 d dry matter)
TPTZ	2,4,6-tripyridyl-s-triazine
